# BCL-2 is dispensable for thrombopoiesis and platelet survival

**DOI:** 10.1038/cddis.2015.97

**Published:** 2015-04-16

**Authors:** M A Debrincat, I Pleines, M Lebois, R M Lane, M L Holmes, J Corbin, C J Vandenberg, W S Alexander, A P Ng, A Strasser, P Bouillet, M Sola-Visner, B T Kile, E C Josefsson

**Affiliations:** 1 The Walter and Eliza Hall Institute of Medical Research, 1G Royal Parade, Parkville, VIC, Australia; 2 Department of Medical Biology, The University of Melbourne, 1G Royal Parade, Parkville, VIC, Australia; 3 Boston Children's Hospital, Division of Newborn Medicine, Boston, MA, USA

## Abstract

Navitoclax (ABT-263), an inhibitor of the pro-survival BCL-2 family proteins BCL-2, BCL-X_L_ and BCL-W, has shown clinical efficacy in certain BCL-2-dependent haematological cancers, but causes dose-limiting thrombocytopaenia. The latter effect is caused by Navitoclax directly inducing the apoptotic death of platelets, which are dependent on BCL-X_L_ for survival. Recently, ABT-199, a selective BCL-2 antagonist, was developed. It has shown promising anti-leukaemia activity in patients whilst sparing platelets, suggesting that the megakaryocyte lineage does not require BCL-2. In order to elucidate the role of BCL-2 in megakaryocyte and platelet survival, we generated mice with a lineage-specific deletion of *Bcl2*, alone or in combination with loss of *Mcl1* or *Bclx*. Platelet production and platelet survival were analysed. Additionally, we made use of BH3 mimetics that selectively inhibit BCL-2 or BCL-X_L_. We show that the deletion of BCL-2, on its own or in concert with MCL-1, does not affect platelet production or platelet lifespan. Thrombocytopaenia in *Bclx*-deficient mice was not affected by additional genetic loss or pharmacological inhibition of BCL-2. Thus, BCL-2 is dispensable for thrombopoiesis and platelet survival in mice.

Platelets are anucleate blood cells that play essential roles in haemostasis, wound healing and a range of other processes, including inflammation and immunity.^1^ They are produced by megakaryocytes, large polyploid cells that develop primarily in the bone marrow, spleen and foetal liver.^2^ Recent work has demonstrated that the survival of megakaryocytes and platelets is governed by the BCL-2 family proteins.^3^ Both cell types possess a classical BAK/BAX-mediated intrinsic apoptosis pathway that must be restrained in order for them to develop and survive.

In platelets, BCL-X_L_ is the critical pro-survival BCL-2 family member required to keep BAK and BAX in check. The first evidence of this came from Wagner *et al.*,^4^ who reported severe thrombocytopaenia in mice after *MMTV-Cre*-mediated deletion of *Bclx* in the haematopoietic system, skin and various secretory tissues. It has since been shown that megakaryocyte-restricted deletion of *Bclx* in mice reduces platelet lifespan from ~5 days to ~5 h, with a concomitant decrease in platelet counts to ~2% of wild-type levels.^5, 6^ Pharmacological inhibition of BCL-X_L_ with the BH3 mimetics ABT-737^7^ or Navitoclax (ABT-263)^8^ (which both also inhibit BCL-2 and BCL-W) triggers BAK/BAX-mediated platelet apoptosis.^9, 10, 11^ As a result, these drugs cause dose-dependent thrombocytopaenia in mice, dogs and humans.^9, 11, 12, 13, 14^ Indeed, thrombocytopaenia is the dose-limiting toxicity for Navitoclax.^12, 13, 14^ This fact provided additional impetus for the development of agents that specifically target BCL-2, beginning with ABT-199,^15^ a BCL-2-selective antagonist currently in clinical trials for the treatment of a range of haematological malignancies including chronic lymphocytic leukaemia, non-Hodgkin's lymphoma, follicular lymphoma, mantle cell lymphoma, multiple myeloma and acute myeloid leukaemia. ABT-199 has already shown very promising anti-tumour activity, with little to no impact on platelet counts.^15, 16^ These data suggest that BCL-2 is dispensable for the development and survival of platelets.

In megakaryocytes, BCL-X_L_ is also critical for survival. Although not absolutely required for their growth and maturation, BCL-X_L_ is essential for megakaryocytes to proceed safely through pro-platelet formation and platelet shedding.^5^ In addition to BCL-X_L_, megakaryocytes also depend on the pro-survival activity of MCL-1. Conditional deletion of *Mcl1* alone has no effect on this lineage. In contrast, combined megakaryocyte-specific loss of *Bclx* and *Mcl1* results in the failure of megakaryopoiesis, systemic haemorrhage and embryonic lethality.^5, 17, 18^ These defects are rescued by deletion of *Bak* and *Bax*.^18^

Consistent with the genetic studies, administration of ABT-737 to *Mcl1*^*Pf4Δ/Pf4Δ*^ mice, which lack MCL-1 in megakaryocytes and platelets, induces acute, fulminant BAK/BAX-dependent megakaryocyte apoptosis. Given that, in addition to BCL-X_L_, ABT-737 also targets BCL-2,^7^ these data suggested that BCL-2 might also contribute to the development and survival of the megakaryocyte lineage. This is supported by recent studies demonstrating that neonatal human platelets contain increased levels of BCL-2 relative to adult counterparts,^19^ and that platelet lifespan is extended in transgenic mice expressing BCL-2 under the control of the pan-haematopoietic *Vav* promoter.^20^ In light of these observations, and intense ongoing activity surrounding the development of novel BH3 mimetics,^21^ we set out to elucidate the role of BCL-2 in megakaryocytes and platelets. Mice with a megakaryocyte-specific deletion of *Bcl2*, either alone or in combination with deletion of *Mcl1* or *Bclx,* were generated. The effect of these mutations, and of BCL-2 or BCL-X_L_-selective BH3 mimetics, on the megakaryocyte lineage was assessed.

## Results

### Platelet production and platelet lifespan are normal in the absence of BCL-2

**Figure 1:**
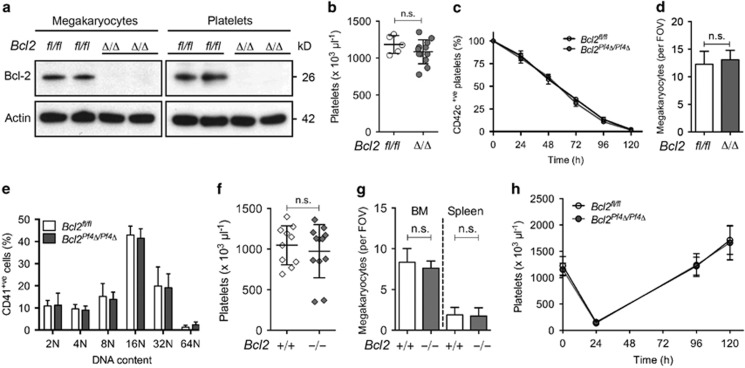
Loss of BCL-2 does not affect platelet survival or platelet production. (**a**) Western blot analysis of protein lysates from platelets and bone marrow-derived megakaryocytes from *Bcl2*^*fl/fl*^ and *Bcl2*^*Pf4Δ/Pf4Δ*^ mice. Bone marrow progenitor cells were cultured in thrombopoietin (TPO), and mature megakaryocytes were purified on a BSA gradient. Probing for actin was used as a control for protein loading. Each lane represents platelets from an individual mouse. (**b**) Platelet counts in *Bcl2*^*Pf4Δ/Pf4Δ*^ and *Bcl2*^*fl/fl*^ control mice at 7–10 weeks of age. Each symbol represents an individual mouse. (**c**) Platelet survival curves in *Bcl2*^*Pf4Δ/Pf4Δ*^ and *Bcl2*^*fl/fl*^ control mice. Platelets were labelled via i.v. injection of a DyLight 488-conjugated anti-CD42c (GPIbβ) Ab. *n*=3 *Bcl2*^*Pf4Δ/Pf4Δ*^ and *n*=5 *Bcl2*^*fl/fl*^ mice. Time 0 (100%) was set at 1 h post injection. (**d**) Morphologically recognisable bone marrow megakaryocytes in H&E-stained sternum sections from *Bcl2*^*Pf4Δ/Pf4Δ*^ and *Bcl2*^*fl/fl*^ control mice. Field of view (FOV). *n*=22 *Bcl2*^*fl/fl*^; *n*=11 *Bcl2*^*Pf4Δ/Pf4Δ*^ mice. (**e**) Ploidy distribution profile of CD41^+^ bone marrow cells in *Bcl2*^*Pf4Δ/Pf4Δ*^ and *Bcl2*^*fl/fl*^ control mice, as determined by flow cytometry. *n*=3 *Bcl2*^*fl/fl*^; *n*=4 *Bcl2*^*Pf4Δ/Pf4Δ*^ mice. (**f**) Platelet counts in *Bcl2*^*-/-*^ and *Bcl2*^*+/+*^ mice at 2–5 weeks of age. Each symbol represents an individual mouse. (**g**) Morphologically recognisable bone marrow (BM) and spleen megakaryocytes in H&E-stained sections from *Bcl2*^*−/−*^ and *Bcl2*^*+/+*^ mice at 1–4 weeks of age. *n*=5 *Bcl2*^*+/+*^, *n*=6 *Bcl2*^*-/-*^mice. (**h**) Platelet counts in mice treated with anti-platelet serum. *n*=5–6 *Bcl2*^*fl/fl*^, *n*=4 *Bcl2*^*Pf4Δ/Pf4Δ*^ mice per time point (except 96 h, *n*=2). Data are presented as mean±S.D.

Mice lacking BCL-2 in the megakaryocytic lineage were generated by crossing animals carrying a floxed allele of *Bcl2*^22^ with *Pf4-Cre* transgenic animals.^23^ *Bcl2*^*Pf4Δ/Pf4Δ*^ mice were born at the expected Mendelian ratios, and were outwardly healthy. Deletion of BCL-2 in bone marrow-derived megakaryocytes and washed platelets was confirmed by western blotting (Figure 1a). Peripheral blood platelet counts (Figure 1b) and platelet survival (Figure 1c) in adult *Bcl2*^*Pf4Δ/Pf4Δ*^ mice were comparable with control animals. Megakaryocyte numbers and ploidy in bone marrow of *Bcl2*^*Pf4Δ/Pf4Δ*^ mice were normal (Figures 1d and e). Additionally, we assessed platelet and megakaryocyte counts in young (1–5-week old) mice with a constitutive deletion of *Bcl2*.^24^ Despite their various phenotypic abnormalities, including kidney polycystic disease, growth retardation and lymphopaenia, blood platelet counts (Figure 1f) and bone marrow and spleen megakaryocyte numbers (Figure 1g) in *Bcl2*^*−/−*^ mice were comparable with those of wild-type controls. Together, these results indicated that BCL-2 is dispensable for steady state platelet production. To establish whether this is also the case under conditions of stress, we induced transient thrombocytopaenia by injecting anti-platelet serum. This typically leads to platelet depletion in wild-type mice within 24 h, followed by recovery and rebound thrombocytosis at ~5 days post injection. *Bcl2*^*Pf4Δ/Pf4Δ*^ and *Bcl2*^*fl/fl*^ mice responded similarly to anti-platelet serum treatment (Figure 1h), indicating that even under conditions of stress thrombopoiesis, BCL-2 is dispensable for the development and survival of megakaryocytes and platelets.

### Combined loss of BCL-2 and MCL-1 does not affect platelet production or platelet survival

**Figure 2:**
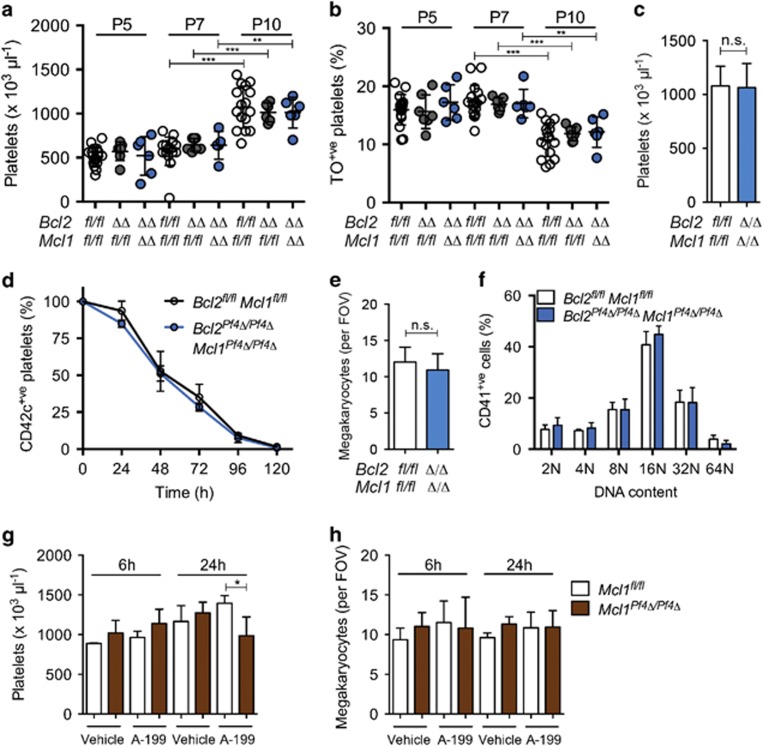
Combined deletion of BCL-2 and MCL-1 does not affect platelet production. (**a**) Platelet counts and (**b**) % reticulated thiazole orange (TO)-positive platelets in floxed control, *Bcl2*^*Pf4Δ/Pf4Δ*^ and *Bcl2*^*Pf4Δ/Pf4Δ*^ *Mcl1*^*Pf4Δ/Pf4Δ*^ neonatal mice on perinatal day (P) 5, 7 and 10. Each symbol represents an individual mouse. (**c**) Platelet counts in adult *Bcl2*^*Pf4Δ/Pf4Δ*^ *Mcl1*^*Pf4Δ/Pf4Δ*^ and *Bcl2*^*fl/fl*^ *Mcl1*^*fl/fl*^ control mice at 7–10 weeks of age. *n*=6 *Bcl2*^*Pf4Δ/Pf4Δ*^ *Mcl1*^*Pf4Δ/Pf4Δ*^; *n*=20 *Bcl2*^*fl/fl*^ *Mcl1*^*fl/fl*^. (**d**) Platelet survival curves in *Bcl2*^*Pf4Δ/Pf4Δ*^ *Mcl1*^*Pf4Δ/Pf4*^ and floxed control mice. Platelets were labelled via i.v. injection of a Dylight 488-conjugated anti-CD42c mAb. *n*=6 mice per genotype. Time 0 (100%) was set at 8 h post injection. (**e**) Numbers of morphologically recognisable megakaryocytes in H&E-stained bone marrow sections. *n*=4 *Bcl2*^*fl/fl*^ *Mcl1*^*fl/fl*^; *n*=7 *Bcl2*^*Pf4Δ/Pf4Δ*^ *Mcl1*^*Pf4Δ/Pf4*^ mice. (**f**) Ploidy distribution profile of CD41^+^ bone marrow cells, as determined by flow cytometry. *n*=4 *Bcl2*^*Pf4Δ/Pf4Δ*^ *Mcl1*^*Pf4Δ/Pf4Δ*^, *n*=2 *Bcl2*^*fl/fl*^ *Mcl1*^*fl/fl*^. Platelet (**g**) and bone marrow megakaryocyte counts (**h**) 6 and 24 h post oral administration of the BCL-2 selective BH3 mimetic ABT-199 (A-199) 100 mg/kg or vehicle control. *n*=3–5 mice per group, except *Mcl1*^*fl/fl*^ 6 h vehicle where *n*=2. *P<0.05; **P<0.005; ***P<0.001. Data are presented as mean±S.D.

We and others have previously shown that platelet production and platelet counts are normal in *Mcl1*^*Pf4Δ/Pf4Δ*^ mice, whereas combined deletion of *Mcl1* and *Bclx* in megakaryocytes results in haemorrhage and embryonic lethality.^17, 18^ To examine any potential functional redundancy between BCL-2 and MCL-1 in the megakaryocyte lineage, we conditionally deleted both of the genes encoding these proteins. We began by measuring body weight, platelet counts and the proportion of reticulated platelets in newborn mice 5–10 days after birth. *Bcl2*^*Pf4Δ/Pf4Δ*^ *Mcl1*^*Pf4Δ/Pf4Δ*^ double knockout animals gained weight at a rate comparable with control littermates (Supplementary Figure 1a). Platelet counts significantly increased from day 7 to 10 in all mice with no differences between genotypic classes (Figure 2a). This correlated with a reduction in the proportion of reticulated (thiazole orange-stained) platelets (Figure 2b). In adult *Bcl2*^*Pf4Δ/Pf4Δ*^ *Mcl1*^*Pf4Δ/Pf4Δ*^ mice, platelet counts and platelet survival were equivalent to those of control littermates (Figures 2c and d). We confirmed efficient deletion of BCL-2 and MCL-1 in bone marrow-derived BSA-gradient-purified megakaryocytes by western blotting (Supplementary Figure 1b). Bone marrow megakaryocyte numbers and ploidy were normal in *Bcl2*^*Pf4Δ/Pf4Δ*^ *Mcl1*^*Pf4Δ/Pf4Δ*^ mice, and these animals exhibited a wild-type response to anti-platelet serum-induced thrombocytopaenia (Figures 2e and f, Supplementary Figure 1c). Consistent with these observations, administration of a single dose of the BCL-2-selective antagonist ABT-199 (100 mg/kg) to *Mcl1*^*Pf4Δ/Pf4Δ*^ and *Mcl1*^*fl/fl*^ mice did not markedly affect blood platelet or bone marrow megakaryocyte numbers 6 and 24 h post injection (Figures 2g and h). Although a statistically significant reduction in platelet counts at 24 h post treatment was observed in *Mcl1*^*Pf4Δ/Pf4Δ*^ mice, relative to *Mcl1*^*fl/fl*^ counterparts (Figure 2g), the numbers were in the normal range (987.6±235.1 × 10^3^/*μ*l) and not significantly different from vehicle-treated *Mcl1*^*Pf4Δ/Pf4Δ*^ animals. Collectively, these results demonstrated that the combined functions of BCL-2 and MCL-1 are dispensable for platelet production and platelet survival.

### BCL-X_L_ antagonism in MCL-1/BCL-2-deficient mice

**Figure 3:**
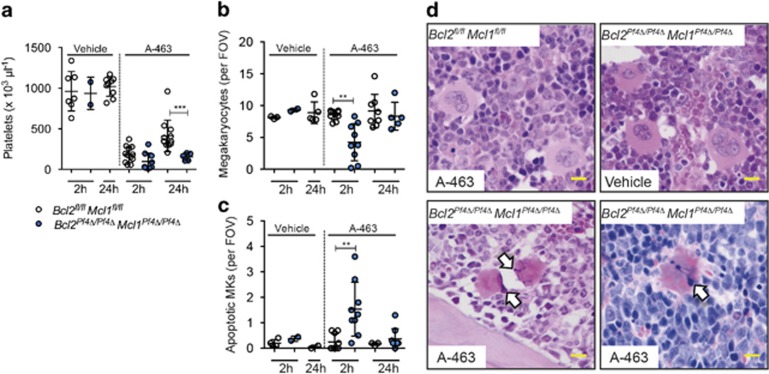
BCL-X_L_ inhibition leads to thrombocytopaenia and apoptosis of MCL-1/BCL-2 deficient megakaryocytes *in vivo*. Platelet (**a**) and bone marrow megakaryocyte counts (**b**) 2 and 24 h post i.p. administration of the BCL-X_L_-selective BH3 mimetic drug A-1155463.7 (A-463) 5 mg/kg or vehicle in *Bcl2*^*fl/fl*^ *Mcl1*^*fl/fl*^ and *Bcl2*^*Pf4Δ/Pf4Δ*^ *Mcl1*^*Pf4Δ/Pf4*^ mice. Each symbol represents an individual mouse. **P<0.005; ***P<0.001. Data are presented as mean±S.D. (**c**) Apoptotic bone marrow megakaryocytes with pyknotic nuclei in H&E sections. (**d**) Representative images of H&E-stained spleen (lower right panel) and bone marrow (all other panels) megakaryocytes 2 h post A-463 or vehicle treatment. Scale bar: 20 *μ*m. Pyknotic nuclei are indicated by white arrows

The recent development of BCL-X_L_-specific BH3 mimetic compounds^25, 26, 27^ enabled us to determine the combined effect of BCL-X_L_, BCL-2 and MCL-1 inhibition on the megakaryocyte lineage *in vivo*. *Bcl2*^*Pf4Δ/Pf4Δ*^ *Mcl1*^*Pf4Δ/Pf4Δ*^ double knockout and control littermates were treated with a single dose of the BCL-X_L_-selective antagonist A-1155463.7 (A-463, 5 mg/kg). In *Bcl2*^*fl/fl*^ *Mcl1*^*fl/fl*^ mice, A-463 induced rapid thrombocytopaenia, but had no effect on bone marrow megakaryocyte numbers at 2 and 24 h post injection (Figures 3a, b and d). In contrast, in *Bcl2*^*Pf4Δ/Pf4Δ*^ *Mcl1*^*Pf4Δ/Pf4Δ*^ animals, acute thrombocytopaenia was observed (Figure 3a). Additionally, apoptotic megakaryocytes with pyknotic nuclei were apparent in the bone marrow and spleen 2 h post administration (Figures 3c and d). Megakaryocyte numbers were reduced, and consistent with this, platelet counts were lower in A-463-treated *Bcl2*^*Pf4Δ/Pf4Δ*^ *Mcl1*^*Pf4Δ/Pf4Δ*^ mice compared with floxed control animals 24 h post injection (Figures 3a and b). These results prompted us to explore functional redundancy between BCL-2 and BCL-X_L_ in the control of cell survival in megakaryocytes and platelets.

### Inhibition of BCL-2 does not exacerbate thrombocytopaenia in BCL-X_L_-deficient mice

**Figure 4:**
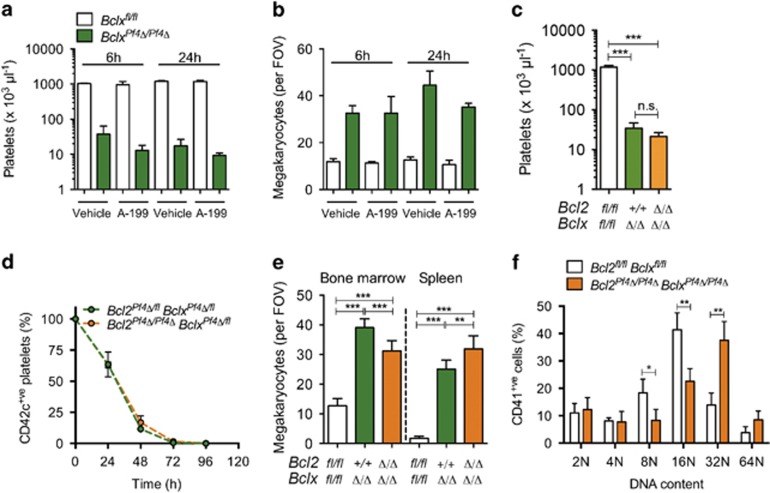
Thrombocytopaenia in *Bclx*-deficient mice is not exacerbated by BCL-2 inhibition. Platelet (**a**) and bone marrow megakaryocyte counts (**b**) 6 and 24 h post oral administration of the BCL-2-selective BH3 mimetic ABT-199 (A-199) 100 mg/kg or vehicle control. *n*=3–4 mice per group, except *Bclx*^*Pf4Δ/Pf4Δ*^ 24 h vehicle where *n*=2. Platelet counts are shown on a log scale. (**c**) Platelet counts (log scale) in *Bcl2*^*Pf4Δ/Pf4Δ*^ *Bclx*^*Pf4Δ/Pf4Δ*^, *Bcl2*^*+/+*^ *Bclx*^*Pf4Δ/Pf4Δ*^ and floxed control mice at 7–10 weeks of age. (**d**) Platelet survival curves in *Bcl2*^*Pf4Δ/Pf4Δ*^ *Bclx*^*Pf4Δ/fl*^ and *Bcl2*^*Pf4Δ/fl*^ *Bclx*^*Pf4Δ/fl*^ mice. Platelets were labelled via i.v. injection of a DyLight 488-conjugated anti-CD42c mAb. *n*=4 mice per genotype. Time 0 (100%) was set at 1 h post injection. (**e**) Numbers of morphologically recognisable megakaryocytes in H&E-stained bone marrow and spleen sections. *n*=8–17 mice per genotype. (**f**) Ploidy distribution profile of CD41^+^ bone marrow cells, as determined by flow cytometry. *n*=4 mice per genotype. *P<0.05; **P<0.005; ***P<0.001. Data represent mean±S.D.

We administered the BCL-2-selective antagonist ABT-199 (100 mg/kg) to *Bclx*^*Pf4Δ/Pf4Δ*^ and floxed control littermates by oral gavage and measured platelet and megakaryocyte counts 6 and 24 h post treatment. Conditional deletion of BCL-X_L_ in the megakaryocytic lineage causes a profound shortening in platelet lifespan, consequent thrombocytopaenia and reactive megakaryocytosis.^5^ Consistent with this, vehicle-treated *Bclx*^*Pf4Δ/Pf4Δ*^ mice exhibited thrombocytopaenia and increased megakaryocyte counts (Figures 4a and b). No significant exacerbation of these deficient platelet and megakaryocyte numbers were observed when *Bclx*^*Pf4Δ/Pf4Δ*^ mice were treated with ABT-199 (Figures 4a and b). To complement the pharmacological approach, we generated *Bcl2*^*Pf4Δ/Pf4Δ*^ *Bclx*^*Pf4Δ/Pf4Δ*^ double knockout mice. Similar to their *Bclx*^*Pf4Δ/Pf4Δ*^ littermates, *Bcl2*^*Pf4Δ/Pf4Δ*^ *Bclx*^*Pf4Δ/Pf4Δ*^ animals were present at weaning at ~75% of the number expected (25 observed/33 expected). Platelet counts in adult *Bcl2*^*+/+*^ *Bclx*^*Pf4Δ/Pf4Δ*^ and *Bcl2*^*Pf4Δ/Pf4Δ*^ *Bclx*^*Pf4Δ/Pf4Δ*^ mice were identical (Figure 4c). In *Bclx* heterozygotes (*Bclx*^*Pf4Δ/fl*^), deletion of one or both alleles of *Bcl2* had no additional impact on platelet numbers and lifespan (Figure 4d). Megakaryocyte counts in bone marrow and spleen were increased in adult *Bcl2*^*+/+*^ *Bclx*^*Pf4Δ/Pf4Δ*^ and *Bcl2*^*Pf4Δ/Pf4Δ*^ *Bclx*^*Pf4Δ/Pf4Δ*^ mice relative to control animals (Figure 4e). We did observe a modest, but statistically significant, decrease in bone marrow megakaryocytes, and an increase in splenic megakaryocytes in *Bcl2*^*Pf4Δ/Pf4Δ*^ *Bclx*^*Pf4Δ/Pf4Δ*^animals relative to *Bcl2*^*+/+*^ *Bclx*^*Pf4Δ/Pf4Δ*^ counterparts. However, these mice were bred in different colonies, which likely explains the minor differences. As expected, and similar to *Bclx*^*Pf4Δ/Pf4Δ*^ mice, megakaryocyte ploidy was increased in *Bcl2*^*Pf4Δ/Pf4Δ*^ *Bclx*^*Pf4Δ/Pf4Δ*^ animals (Figure 4f).

### Pharmacological inhibition of BCL-X_L_, but not BCL-2, induces megakaryocyte apoptosis

**Figure 5:**
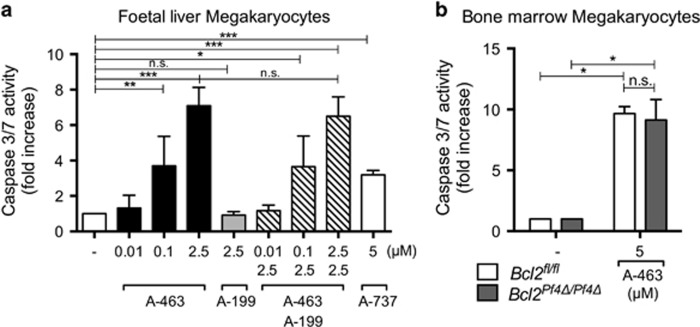
BCL-X_L_ inhibition induces cell death in megakaryocytes *in vitro*. (**a**) Caspase-3/7 activity expressed as fold-increase in wild-type foetal liver-derived BSA gradient-purified megakaryocytes 5 h post treatment with A-1155463.7 (A-463) 0.01–2.5 *μ*M, ABT-199 (A-199) 2.5 *μ*M, ABT-737 (A-737) 5 *μ*M or vehicle (DMSO) was assessed by using the Caspase-3/7 Glo (Promega) kit. *n*=4–5 biological replicates, three independent experiments. (**b**) Caspase-3/7 activity expressed as fold-increase in bone marrow-derived BSA gradient-purified *Bcl2*^*Pf4Δ/Pf4Δ*^ and *Bcl2*^*fl/fl*^ megakaryocytes 5 h post treatment with A-463 (5 *μ*M) or vehicle (DMSO) was assessed by using the Caspase-3/7 Glo kit. Data shown are representative of two independent experiments. *P<0.05; **P<0.005; ***P<0.001. Data represent mean±S.D.

The results from our *in vivo* studies suggested that genetic or pharmacological antagonism of BCL-2 has no adverse effect on megakaryopoiesis. To test this directly in isolated primary cells, we treated foetal liver or bone marrow-derived mouse megakaryocytes with ABT-737, ABT-199 or A-463. It has been previously reported that in cultured primary mouse megakaryocytes, deletion of *Bcl-x* or treatment with ABT-737 triggers loss of cell viability, Caspase-3/7 activity and a failure of proplatelet formation.^5^ In line with these data, we found that the BCL-X_L_-selective inhibitor A-463 induced dose-dependent Caspase-3/7 activation in wild-type foetal liver-derived megakaryocytes (Figure 5a). Consistent with its increased potency against BCL-X_L,_^27^ A-463 treatment at a concentration of 2.5 *μ*M induced more caspase activity in megakaryocytes than ABT-737 at 5 *μ*M. In contrast, treatment with the BCL-2 antagonist ABT-199 (2.5 *μ*M) had no effect. Combination treatment with A-463 and ABT-199 did not amplify Caspase-3/7 activity beyond that seen with A-463 treatment alone. Similarly, loss of *Bcl2* did not render bone marrow-derived megakaryocytes more sensitive to A-463 (Figure 5b).

## Discussion

In this study, we analysed platelet production in mice with a megakaryocytic lineage-specific deletion of *Bcl2* alone or in combination with deletion of *Mcl1* or *Bclx.* In addition, selective BH3 mimetics inhibiting BCL-2 or BCL-X_L_ were assessed. Our genetic and pharmacological studies demonstrate that BCL-2 is dispensable for platelet production both at steady state and under conditions of stress. Moreover, platelet survival *in vivo* was not affected by genetic loss of BCL-2 or its pharmacological inhibition. This aligns with initial reports on the effects of ABT-199 in patients,^15, 16^ and bodes well for the clinical development of BCL-2 antagonists. Our experiments confirm the importance of BCL-X_L_ and the ancillary role of MCL-1 in maintaining megakaryocyte viability and BCL-X_L_ in sustaining platelet survival.^5^

Our data indicate that BCL-2 is dispensable for platelet survival in adults, and in neonates as well. The latter findings are consistent with a recent study from Liu and colleagues,^19^ which demonstrated that although neonatal platelets display elevated BCL-2 protein levels and extended survival compared with adult platelets, BCL-2 was not the primary molecule facilitating this effect. We have further demonstrated that conditional deletion of *Bcl2*, on its own or in concert with *Mcl1*, had no impact on neonatal platelet counts.

BCL-2 has recently been linked to myeloid progenitor cell survival.^28^ As Cre-dependent recombination utilising the *Pf4-Cre* transgenic mouse is restricted to megakaryocytes, platelets^23^ and a small fraction of late megakaryocyte progenitors,^29^ we could not use this model to address the role of BCL-2 in the earliest stages of megakaryopoiesis. However, mice carrying the constitutive deletion of *Bcl2* did afford this opportunity. Germline loss of BCL-2 leads to polycystic kidney disease, lymphopaenia, grey fur (due to premature death of melanocytes), growth retardation and early mortality.^24, 30, 31^ Even with the complex co-morbidities of this model, our results show that young *Bcl2*^*−/−*^ and control mice exhibited similar megakaryocyte and platelet counts, indicating that loss of BCL-2 in progenitor cells does not significantly affect the megakaryocytic lineage at steady state in young animals. Although it is increasingly clear that ABT-199 used as a single agent within the therapeutic window will not kill platelets and megakaryocytes, potential effects on progenitor cells with subsequent thrombocytopaenia may become apparent when used in combination with certain chemotherapeutics. This is pertinent as clinical trials are currently evaluating the safety and efficacy of ABT-199 in combination with proteasome inhibitors in multiple myeloma,^32^ hypomethylating agents in acute myeloid leukaemia,^33^ and alkylating or antimitotic drugs in non-Hodgkin's lymphoma, chronic lymphocytic leukaemia and follicular lymphoma^34, 35^ known to cause thrombocytopaenia through bone marrow suppression or inhibition of proplatelet formation.^36, 37^

Despite dose-limiting thrombocytopaenia, BCL-X_L_ antagonism has shown encouraging results in certain solid tumours.^12^ ^38^ Combination trials with Navitoclax and kinase inhibitors (MEK, RAF and BRAF) are underway for advanced or metastatic solid tumours, including small-cell lung, colon, pancreatic, rectal and liver cancer. Limited information is available on the effects of this group of kinase inhibitors on megakaryocytes and platelets, although MEK inhibition has been associated with thrombocytopaenia.^39^ Hence, for combination treatments using BCL-X_L_ antagonists and kinase inhibitors, it will be imperative to closely monitor platelet counts, as both platelet production and platelet survival may be affected. With the recent development of BCL-X_L_–specific BH3 mimetics,^25, 26, 27^ there is a need to find means of sustaining platelet counts in order to allow safe dose escalation without increased risk of bleeding.^40^ Although BCL-2 is not required for platelet survival, we recently showed that overexpression of BCL-2 in blood cells extends platelet lifespan in adult mice,^20^ similarly to that observed in animals lacking the essential mediators of intrinsic apoptosis, BAK and BAX.^5, 9^ One could imagine that one approach to facilitating the safe administration of BCL-X_L_ antagonists might be transfusion of platelets either overexpressing BCL-2, or lacking BAK/BAX, thus rendering them resistant to BCL-X_L_ inhibition. Recent advances in this field of research, including development of human-induced pluripotent stem cell-derived megakaryocytes generating platelets,^41^ HLA-universal platelets,^42^ synthetic micro-vessels^43^ and a novel microfluidic bioreactor design enabling *ex vivo* platelet production,^44^ might allow such manipulation in the not-too-distant future.

## Materials and Methods

### Mice

*Bcl2*^-/-,24^ *Bcl2* floxed,^22^ *Mcl1* floxed,^9^ *Bclx* floxed^45^ and *Pf4-Cre*^23^ mice have been previously described. All mouse lines had been backcrossed onto the C57BL/6 background for at least 10 generations prior to this study. Mice were 7–12-weeks old or as otherwise stated. All animal experiments complied with the regulatory standards of, and were approved by, the Walter and Eliza Hall Institute (WEHI) Animal Ethics Committee.

### Materials

Dimethyl sulfoxide (DMSO) and propidium iodide were from Sigma-Aldrich, St. Louis, MO, USA. ABT-737^7^ was provided by Abbott Laboratories, Abbott Park, IL, USA. ABT-199^15^ and A-1155463.7^27^ were provided by AbbVie, North Chicago, IL, USA. The enhanced chemiluminescence system was from Merck Millipore (Kilsyth, VIC, Australia), the protease inhibitor cocktail, Complete, was from Roche (Basel, Switzerland), 4–12% Bis-Tris gels (NuPAGE) were from Invitrogen Life technologies (Carlsbad, CA, USA), and calibration beads 3.5–4.0 μm were from Spherotech Inc (Lake Forest, IL, USA). Antibodies used for immunoblotting included: mouse anti-BCL-2 (Clone 7; recognises mouse, rat, chicken, dog and human) from BD Transduction Laboratories (Franklin Lakes, NY, USA), rat anti-MCL-1 (clone 19C4-15; recognises mouse and human, WEHI mAb Facility, Parkville, VIC, Australia) and anti-actin-HRP from Santa Cruz Biotechnology (Dallas, TX, USA). Antibodies used for flow cytometry included: X488 from Emfret Analytics GmbH and Co. KG (Eibelstadt, Germany); fluorescently conjugated anti–mouse CD41 (MWReg30) from BD. Anti-platelet serum was purchased from Cedarlane (Burlington, Ontario, Canada).

### Haematology

Automated cell counts were performed on blood collected by cardiac puncture or from the retro-orbital plexus into Microtainer tubes containing EDTA (Sarstedt, Ingle Farm, SA, Australia), using an Advia 2120 haematological analyser (Siemens, Munich, Germany). Megakaryocytes were counted manually in sections of sternum and spleen stained with haematoxylin and eosin (H&E) with a minimum of 10 high-power fields ( × 200) analysed. Images were acquired on a Nikon Eclipse E600 microscope equipped with AxioCam MRc5 (Zeiss, Oberkochen, Germany) and AxioVision 4.8. Scale bars were inserted with Image J. Acute thrombocytopaenia was induced by injection of anti-platelet serum and assessed as described.^46^

### Primary megakaryocyte culture

Foetal livers were removed at embryonic day (E) 13.5 and transferred into Dulbecco's modified Eagle's medium (high glucose version) with 10% foetal calf serum (Gibco, Paisley, UK). Bone marrow was flushed into Dulbecco's modified Eagle's medium with 2% foetal calf serum. The cells were lineage-depleted by incubation with a mix of biotinylated antibodies (CD4, CD2, CD3, CD5, CD8, Ter119, B220, CD19, Gr-1, Ly6G, F4/F8, CD127; WEHI mAb Facility) in KDS-BSS 2% foetal calf serum, followed by anti-biotin magnetic microbeads (Miltenyi Biotec, Bergisch Gladbach, Germany) and MAC LS columns (Miltenyi Biotec) in EDTA-KDS-BSS 0.5% foetal calf serum. Single cell suspensions were cultured for 3–5 days at 5 × 10^5^ cells per ml in serum-free medium^47^ supplemented with 100 ng/ml murine thrombopoietin (WEHI) at 37 °C, 5% CO_2_, and mature megakaryocytes purified using a BSA gradient as described.^5^

### Megakaryocyte ploidy

Bone marrow was harvested from femora of 8–10-week-old mice into 1 ml of CATCH medium and megakaryocyte ploidy was studied by staining with CD41-FITC mAb and propidium iodide, as described.^5^

### Caspase activity assay

BSA gradient-purified megakaryocytes (3 × 10^4^ cells per ml in serum-free medium with TPO) were seeded into 96-well plates and then incubated at 37 °C, 5% CO_2_ with or without the addition of ABT-737 (5 *μ*M), ABT-199 (2.5 *μ*M) or A-1155463.7 (0.01–5 *μ*M). Caspase-Glo 3/7 reagents (Promega, Madison, WI, USA) were added to megakaryocytes after 5 h of treatment. The luminescence of each sample was determined in a plate-reading LumiSTAR Galaxy luminometer (BMG Labtech, Ortenberg, Germany).

### Platelet preparation

Murine blood was obtained by cardiac puncture into 0.1 volume of Aster-Jandl anticoagulant (85 mM sodium citrate, 69 mM citric acid, and 20 mg/ml glucose, pH 4.6).^48^ Platelet-rich plasma was obtained by centrifugation at 125 × *g* for 8 min, followed by centrifugation of the supernatant buffy coat at 125 × *g* for 8 min. Platelets were washed by two sequential centrifugations at 860 × *g* for 5 min in 140 mM NaCl, 5 mM KCl, 12 mM trisodium citrate, 10 mM glucose and 12.5 mM sucrose, pH 6.0 (buffer A). The platelet pellet was resuspended in 10 mM Hepes, 140 Mm NaCl, 3 mM KCl, 0.5 mM MgCl_2_, 10 mM glucose and 0.5 mM NaHCO_3_, pH 7.4 (buffer B).

### Platelet turnover studies

Mice were injected i.v. with 0.15 *μ*g/g body weight of X488 (Emfret), a rat-derived IgG against the platelet CD42c (GPIbβ) receptor, and platelet lifespan was measured as previously described.^48^ Platelets were identified in Platelet-rich plasma as being CD41^+^ by flow cytometry and the proportion of X488^+^ platelets remaining at each time point was assessed.

### SDS-PAGE and western blot analysis

Platelets were lysed in NP40 lysis buffer and megakaryocytes were lysed in RIPA buffer. Proteins were separated on 4–12% Bis-Tris gels (NuPAGE; Invitrogen) under reducing conditions, transferred onto Immobilon-P membranes (Micron Separation), and immunoblotted with various Abs (see above), followed by incubation with secondary HRP-conjugated Abs and ECL.

### Platelet counts in newborn mice

Newborn (day P2) mice were given paw tattoos to assist identification. Newborn mice were weighed on P5, 7 and 10 prior to blood collection. Genotyping was performed by PCR using DNA obtained on P2. For blood collection, the anterior facial vein was punctured using a 30-gauge needle. Five microlitres of blood were collected using a micropipette and dispensed into an EDTA blood tube (Sarstedt) with PBS to a total volume of 200 *μ*l. Automated cell counts were performed on blood collected from newborn mice using an Advia 2120 haematological analyser (Siemens). Reticulated platelets in newborn mice were enumerated using staining with thiazole orange.^48^

### *In vivo* administration of BH3 mimetics

Mice were treated via oral gavage with 100 mg/kg ABT-199 administered as a single dose. A stock solution of ABT-199 (10 mg/ml) was diluted in 60% phosal 50 PG (standardised phosphatidylcholine concentrate with at least 50% PC and propylene glycol; Phospholipid GmbH, Cologne, Germany), 30% polyethylene glycol 400 and 10% ethanol. Blood and sterna were collected at 6 and 24 h after treatment. Alternatively, mice were injected i.p. with 5 mg/kg A-1155463.7 administered as a single dose. A stock solution of A-1155463.7 (12.5 mg/ml) in DMSO was diluted by addition of 30% final volume of dosing solution Cremophor ELP:ethanol (ratio 2 : 1) (Cremophor ELP, Sigma-Aldrich) and 5% dextrose in H_2_O (to reach final volume). Blood, sterna and spleens were collected at 2 and 24 h after treatment. Sternum and spleen sections were stained with H&E.

### Statistical analyses

Statistical significance between two treatment groups was analysed using an unpaired Student's *t* test with two-tailed *P* values. One-way ANOVA with the Bonferroni multiple comparison test was applied where appropriate (GraphPad Prism Software Version 6.0b, La Jolla, CA, USA). *P<0.05; **P<0.005; ***P<0.001 or as otherwise stated. Data are presented as mean±S.D.

## Abbreviations

E: embryonic day
FL: foetal liver
H&E: haematoxylin and eosin
TO: thiazole orange
TPO: thrombopoietin
P: perinatal day
