# Supplementary material for: BCL-2 is dispensable for thrombopoiesis and platelet survival
Source: Cell Death Dis. 2015 Apr 16;6(4):e1721–. doi: 10.1038/cddis.2015.97 (PMC4650559; doi:10.1038/cddis.2015.97)
Supplement: Supplementary Information [file cddis201597x1.doc]

**SUPPLEMENTARY INFORMATION**


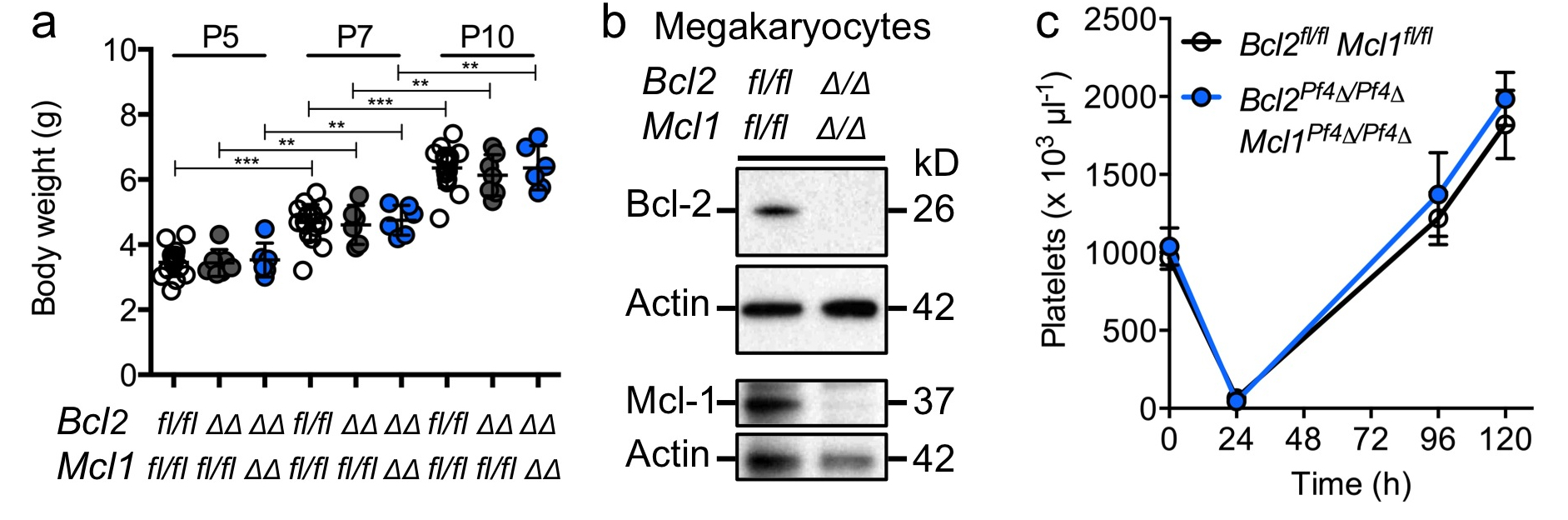


**Supplementary Figure 1** Combined deletion of BCL-2 and MCL-1 does not affect platelet production. (**a**) Body weight in floxed control, *Bcl2^Pf4∆/Pf4∆^* and *Bcl2^Pf4∆/Pf4∆^ Mcl1^Pf4∆/Pf4∆^* neonatal mice on perinatal day (P) 5, 7 and 10. Each symbol represents an individual mouse. Body weight in gram (g). (**b**) Western blot analysis of protein lysates from bone marrow derived megakaryocytes from *Bcl2^fl/fl^ Mcl1^fl/fl^* and *Bcl2^Pf4∆/Pf4∆^ Mcl1^Pf4∆/Pf4∆^* mice. Bone marrow progenitor cells from adult mice were cultured in TPO and mature megakaryocytes were purified on a BSA gradient. Probing for actin was used as a control for protein loading. (**c**) Platelet counts in mice treated with anti-platelet serum. n=4-9 mice per time point and genotype. Data are presented as mean ± s.d.
